# *Perilla frutescens* Sprout Extract Protect Renal Mesangial Cell Dysfunction against High Glucose by Modulating AMPK and NADPH Oxidase Signaling

**DOI:** 10.3390/nu11020356

**Published:** 2019-02-08

**Authors:** Ha-Rim Kim, Seon-Young Kim

**Affiliations:** Jeonju AgroBio-Materials Institute, 54810, Wonjangdong-gil 111-27, Deokjin-gu, Jeonju-si 54810, Korea; Kimseon02@naver.com

**Keywords:** *Perilla frutescens*, murine mesangial cells, diabetic nephropathy, reactive oxygen species (ROS), NADPH oxidase

## Abstract

*Perilla frutescens* (L.) Britt. var. japonica (Hassk.) Hara (PF), is a medical herb of the Lamiaceae family. We have previously reported that the PF sprout extract (PFSE) is effective in treating hyperglycemia. However, the role of PFSE on glomerular mesangial cells (MCs) proliferation and the extracellular matrix (ECM) accumulation in a diabetic condition are still unclear. Therefore, in this study, we have investigated the role of PFSE on cell proliferation and ECM accumulation in murine glomerular MCs (MMCs), cultured under a high glucose (HG) condition. PFSE treatment attenuated HG-induced MMCs proliferation and hypertrophy. Moreover, the HG-induced ECM protein, collagen IV and fibronectin, overexpression was abolished by the PFFSE treatment. In addition, PFSE inhibited reactive oxygen species (ROS) overproduction and NOX2 and NOX4 expression in MMCs under a HG condition. Our data further revealed the involvement of mesangial cell damage in AMP-activated kinase (AMPK) activation. PFSE strongly activated AMPK in MMCs under hyperglycemic conditions. These results suggest that PFSE inhibits HG-medicated MC fibrosis through suppressing the activation of NOX2/4 and the AMPK activation mechanism. PFSE may be useful for the prevention or treatment of diabetic nephropathy.

## 1. Introduction

Diabetic nephropathy (DN) is a leading cause of end-stage renal disease and is associated with increased mortality and morbidity [1]. DN is characterized by the progressive expansion of the mesangium with the accumulation of an extracellular matrix (ECM) in the glomerulus [2]. Glomerular mesangial cells (MCs) proliferation, hypertrophy, and ECM accumulation are hallmarks of diabetic nephropathy [2,3].

Reactive oxygen species (ROS) are ubiquitous, highly reactive short-lived derivatives of oxygen metabolism, produced in all biological systems that react with surrounding molecules at the site of formation. It has been known that ROS, at a low level, play an essential role in multiple cellular signal transduction pathways. However, under conditions of oxidative stress, they contribute to a series of cellular dysfunctions. Several studies support the finding that renal ROS play a central role in mediating renal injury in diabetes [4,5,6]. It has been reported that ROS play an important role in the pathogenesis of renal profibrotic factors in inducing fibroblast proliferation and/or activation [7]. Our previous studies also confirmed that the ROS were increased in mesangial cells (MCs) in high-glucose conditions [8]. NADPH oxidases (NOX) are important sources of ROS involved in both normal physiological functions and oxidative stress [5].

AMP-activated kinase (AMPK) is a cellular energy sensor that exerts diverse biological effects on cell growth, differentiation, and apoptosis, as well as the regulation of glucose and lipid metabolism. AMPK activators mimic the actions of insulin, in terms of gluconeogenesis, repressing glucose production [9,10]. Reduced expression and/or the inhibition of AMPK activity has been involved in the pathogenesis of DN [11]. Numerous cellular and animal experiments report cardiovascular-protective effects of AMPK. Several lines of research indicate that AMPK activation may function as a NOX inhibition [12].

*Perilla frutescens* (L.) Britt. var. japonica (Hassk.) Hara (PF), commonly called perilla or Korean perilla, is a species of perilla belonging to the mint family Lamiaceae. It is a well-known annual herbaceous plant, often used in foods and medicine in Asian countries such as Korea, China, and Japan. This plant is commonly known as “Dlggae” in Korea [13]. Previously, we reported the antioxidant and hypoglycemic effects of the PF sprout extract (PFSE) in pancreatic β-cells and type 2 diabetic animal model [13,14]. However, the protective effect of the PFSE against DN and the underlying mechanism remains elusive. Based on this background, the present study investigated the effect of the PFSE on DN in murine MCs.

## 2. Materials and Methods

### 2.1. Chemicals and Antibodies 

Phosphate-buffered saline (PBS), Dulbecco’s modified Eagle’s medium (DMEM), fetal bovine serum (FBS), and antibiotics (amphotericin B, penicillin, and streptomycin) were purchased from Invitrogen (Carlsbad, CA, USA). Dimethyl sulfoxide (DMSO), 2′,7′-dichlorofluorescein diacetate (DCF-DA), diphenylene iodonium (DPI), and other chemicals were obtained from Sigma (St. Louis, MI, USA). Antibodies were obtained as following sources: anti-phospho-AMPK pAb (sc-33524), anti-AMPK pAb (sc-25729), and anti-NOX4 pAb (sc-30141), anti-NOX2 (gp91phox, sc-5827) pAb, anti-Col I pAb (sc-25974) and anti-fibronectin pAb (sc-9068), and horseradish peroxidase (HRP)-conjugated anti-goat IgG) were purchased from Santa Cruz Biotechnology (SantaCruz, CA, USA) and anti-mouse IgG (#7076), and anti-rabbit IgG (#7074)were purchased from Cell Signaling Technology (Dancers, MA, USA).

### 2.2. Preparation of Samples for Treatment

PF sprouts were obtained from Aeong Association (Jinan, Jeonbuk, Korea) and the extract was prepared by the standard procedure as described previously [13]. In summary, dried sprouts were extracted in 40% aqueous ethanol (EtOH) for 5 h at 70 °C. After filtering the extracts, the solvents were rotary-vacuum evaporated and then freeze dried. The extraction yield from the dry weight of PF sprouts was 15%.

### 2.3. Culture of MMCs 

SV40-transformed MMCs (MES-13) were obtained from the America Type Culture Collection (ATCC; Rockville, MD, USA) and maintained in DMEM containing 5% FBS, 0.25 μg/mL amphotericin B, 100 units/mL penicillin, and 100 units/mL streptomycin at 37 °C in 5% CO_2_, 95% air. Cells were passaged three times per week.

### 2.4. Proliferation Assay 

Cells were seeded at a density of 5 × 10^3^ cells/well in a 96-well plate. When the cells reached 60–70% confluence, the growth medium was aspirated and the wells were rinsed with pre-warmed PBS. Quiescent cells were exposed to a fresh medium with different concentrations of PFSE (0.1~100 μM) or 0.1% DMSO (vehicle control) for 48 h. After incubation, 20 μL of a solution of CellTiter 96 Aqueous One Solution (Promega, Madison, WI, USA) containing MTS [3-(4,5-dimethylthiazol-2-yl)-5-(3-carboxymethoxyphenyl)-2-(4-sulfophenyl)-2H-tetrazolium] and an electron-coupling reagent (phenazine ethosulfate) were added to each well. The plates were incubated for 3 h, during which time the reagent was bio-reduced into a colored formazan product by the intracellular dehydrogenase enzymes of metabolically active cells. The absorbance was measured at 490 (Perkin Elmer Wallac 1420 Victor^2^ Microplate Reader, Whaltam, MA, USA).

### 2.5. Determination of DNA Synthesis

A total of 1 × 10^4^ MMCs/wells were seeded onto 96-well plates and grown to semiconfluence in DMEM containing a normal glucose concentration (NG, 5.5 mmol/L) and 5% FBS for 24 h. Cells were washed once with PBS before growth arresting in DMEM without FBS for 48 h. Quiescent MCs were stimulated with high glucose (HG, 25 mmol/L) and pretreated with different concentrations of PFSE (0.1~100 μg/mL) for 48 h. DNA synthesis was quantified by 5-bromo-2′-deoxyuridine (BrdU) incorporation into proliferating cells over 2 h (Roche Diagnostics, Mannheim, Germany).

### 2.6. Total Protein to Cell Count Ratio 

The ratio of total protein content to cell number is another well-established measure of cellular hypertrophy. To measure this ratio, MMCs were seeded into each well of a six-well plate and were synchronized into quiescence for 12 h in a serum-free medium containing a NG. MCs were stimulated with HG and pretreated with different concentrations of PFSE (10~100 μg/mL) for 48 h. After incubation, cells were trypsinized, scraped off the plate with a rubber policeman, and washed twice in PBS. A small aliquot of cells was used for cell counting of intact viable cells after trypan blue (Sigma-Aldirch, St. Louis, MI, USA) staining to calculate the cell number/protein ratio. The remaining cells were lysed in RIPA buffer (10 mM Tris/HCl (pH 7.4), 150 mM NaCl, 10 mM EGTA, 10 mM EDTA, 10 mM sodium pyrophosphate, 1 mM sodium orthovanadate, 50 mM sodium fluoride, 1% (v/v) triton X-100, 20 μM leupeptin, 1 mM PMSF and 0.15 μM pepstatin), and the total protein content was measured by a Bradford method. Total protein content was expressed as μg protein per 10^5^ cells. These experiments were independently carried out three times.

### 2.7. Measurement of ROS

Generation of intracellular ROS was measured with the fluoroprobe DCF-DA. Briefly, MMCs were incubated for 60 min at 37 °C with 10 μM DCF-DA in absence or presence of PFSE in a dose-dependently. Fluorescence intensity was measured by fluorescence microscopy (NIKON; excitation 488 nm, emission 513 nm). Average intensity for each experimental group of cells was determined using Scion Image Analysis software, and values were expressed as the above control.

### 2.8. Determination of Superoxide Anion Production 

Superoxide anion production by mesangial cells was determined by measuring the superoxide dismutase-inhabitable reduction of cytochrome c with a spectrophotometer as previously described [15]. Briefly, the mesangial cells were grown in 24-well tissue culture plates. The cells were treated with various inhibitors, containing cytochrome c (80 μM) with or without superoxide dismutase (100 μg/mL). The absorbance was then measured with a spectrophotometer at 550 nm. The relative amount of superoxide anions secreted by cells was calculated.

### 2.9. Determination of Reduced Glutathione Levels in MMCs

Reduced glutathione (GSH) content was determined in mesangial cells lysates. GSH content was carried using a glutathione assay kit (Cayman, MI, USA). The kit was based on an enzymatic recycling method, using glutathione reductase for the quantification of GSH.

### 2.10. Determination of NOX Acitivity in MMCs

NOX activity was measured by the lucigenin-enhanced chemiluminescence method as previously described [6]. MMCs were stimulated with glucose for 1 h after treating the PFSE. The cells were washed and homogenized in a lysis buffer containing 20 mM KH_2_PO_4_ (pH 7.0), 1 mM EGTA, 1 mM PMSF, 10 μg/mL aprotinin, and 0.5 μg/mL leupeptin. A 100 μL of homogenate was immediately added to 900 μL of 50 mM phosphate buffer (pH 7.0) containing 1 mM EGTA, 150 mM sucrose, 5 μM lucigenin as an electron acceptor, and 100 μM NADPH. Superoxide production was expressed as the rate of relative chemiluminescence units (RLU) using the Spectra-Max Gemini plate reader (Molecular Devices, Sunnyvale, CA, USA).

### 2.11. Immunoblotting

Protein extraction and immunoblotting of MMCs were performed as previously [16]. Proteins (20 μg per lane) were resolved on a 10% or 12% SDS-PAGE gel and transferred to polyvinylidene difluoride (PVDF, GE Healthcare, Little Chalfont, Buckinghamshire, UK) membranes. Blots were incubated with primary antibodies (1:2500 dilution of each antibody) overnight at 4 °C. The blots were rinsed four times with a blocking buffer and incubated with horseradish peroxidase-conjugated secondary antibodies (1:5000 dilutions of each antibody) for 1 h at room temperature. The binding of the antibodies was visualized using an enhanced chemiluminescence (ECL) system (Bio-Rad, Munich, Germany). Protein concentrations were determined using a Bio-Rad protein assay kit, and known concentrations of bovine serum albumin (BSA) were used as the standard. All immunoreactive signals were analyzed by densitometric scanning (Fuji Photo Film Co., Tokyo, Japan).

### 2.12. Statistical Analysis

The data represented means ± SEM of at least three separate experiments. Statistical comparisons were performed using one-way ANOVA followed by Sigma Plot software (Systat, London, UK). Statistical significance of the difference between groups was determined using the unpaired Student’s *t* test. Differences were considered significant if the P value was less than 0.05.

## 3. Results

### 3.1. Effects of PFSE on High Glucose (HG)-Induced MMCs Growth and Hypertrophy

**Figure 1:**
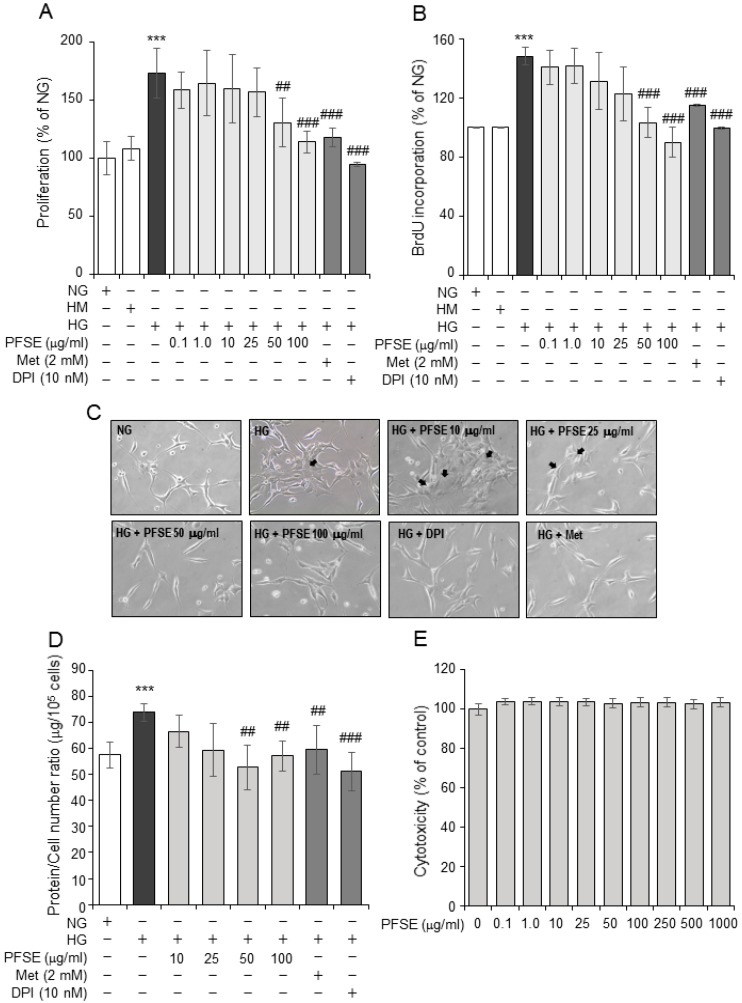
Effect of *Perilla frutescens* sprout extract (PFSE) on a high glucose (HG)-induced proliferation and protein synthesis in murine mesangial cells (MMCs). (**A**) MMCs were treated with HG (25 mM) for 48 h in the absence or presence of PFSE (0.1, 1.0, 10, 25, 50 and 100 μg/mL), Met (metformin, 2 mM), and DPI (diphenylene indonium, 10 nM). After this incubation, cell proliferation was determined with the MTS assay. (**B**) PFSE inhibits HG-induced DNA synthesis in MMCs. DNA synthesis was measured using BrdU cell proliferation assay. (**C**) Representative microscopic images of cells under normal glucose (NG, 5.5 mM) or HG conditions in a presence or absence of PFSE, Met, and DPI. (**D**) The total protein/cell number ratio expressed as μg/10^5^ cells after PFSE treatment for 48 h. (**E**) Cellular cytotoxicity of PFSE in HEK-293T cells. *** *P* < 0.001 vs. normal glucose (NG, 5.5 mM); ^##^ *P* < 0.01, ^###^ *P* < 0.001 vs. HG. Values are expressed as means ± SEM of three independent experiments.

Since one of the earliest renal abnormalities observed after the onset of hyperglycemia often includes the proliferation of MCs, the PFSE was tested for its ability to inhibit MMCs proliferation in a HG (25 mmol/L) condition. HG-stimulated proliferation was evaluated using MTS and BrdU incorporation assay. HG stimulated the growth of MMCs, as compared to a NG (5.5 mmol/L) condition (*P* < 0.01). Diphenylene iodonium (DPI), a NADPH oxidase (NOX) inhibitor, and metformin (Met), an AMPK activator, both significantly inhibited HG induced-proliferation of MMCs (Figure 1A,B), as analyzed by MTS and BrdU incorporation. The PFSE pretreatment with different concentrations (0.1~100 μg/mL) showed significant inhibition of cell growth stimulated by HG (Figure 1A,B), the threshold concentration of the PFSE that caused inhibition of proliferation was 50 μg/mL (130.7 ± 21.2% versus 173.2 ± 21.8%, *P* < 0.05), with a maximal effect at 100 μg/mL (113.9 ± 7.8% vs 173.2 ± 21.8%, *P* < 0.001), as showed in MTS assay (Figure 1A). Similar results were obtained using BrdU incorporation assay (Figure 1B). Unlike HG, the addition of 25 mM mannitol (HM) to the media did not affect the proliferation of MMCs compared with the control, suggesting that the HG-triggered mesangial cell growth is not the results of high osmolality within the media. The morphological change in DN is mesangial cell hypertrophy, which results from an increase in protein synthesis in the early phase of hyperglycemia. As shown in Figure 1C,D, cell morphology and the ratio of the amount of total protein to cell number in MMCs under a HG condition were significantly greater than the NG group (*P* < 0.001). The PFSE treatment abolished the MMCs hypertrophy from 50 μg/mL. The NOX inhibitor and AMPK activator also remarkably blocked the protein synthesis of MMCs. These data suggest that a HG-induced proliferation and hypertrophy is mediated by NOXs and AMPK signaling. In addition, the PFSE treatment did not affect cell growth in MMCs compared with the control at the tested concentration (0.1~1000 μg/mL), which explains an inhibitory effect of PFSE on proliferation and hypertrophy rather than the cytotoxic effect (Figure 1E).

### 3.2. PFSE Reduces Reactive Oxygen Species (ROS) Production and Increases the Activity of Antioxidant Enzymes

**Figure 2:**
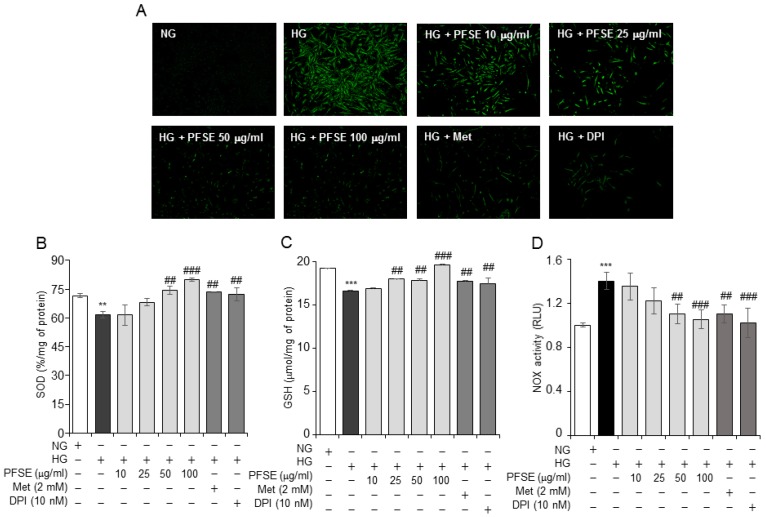
PFSE inhibits HG-induced oxidative stress in MMCs. (**A**) MMCs were treated with HG in the presence or absence of PFSE (10, 25, 50 and 100 μg/mL), Met (2 mM), and DPI (10 nM) for 48 h. Cells were stained with reactive oxygen species-sensitive dye (ROS) 2′,7′-dichlorofluorescein diacetate (DCFDA) and observed under a fluorescence microscope. (**B**) The effects of PFSE on the activity of the antioxidant enzyme, superoxide dismutase (SOD). (**C**) Intracellular reduced glutathione (GSH) in mesangial cells, cultured in 5.5- or 25-mM glucose with or without PFSE, Met, and DPI for 48 h. (**D**) NOX activities were measured as described in Materials and Methods. ** *P* < 0.01, *** *P* < 0.001 vs. NG, ^##^ *P* < 0.01, ^###^ *P* < 0.001 vs. HG. Values are expressed as means ± SEM of three independent experiments.

Oxidative stress could be involved in renal disease. We tested the antioxidative effect of PFSE by HG in MMCs. In our previous results [13], PFSE had an antioxidant effect in pancreatic β-cells. As showed in Figure 2A, HG stimulated intracellular ROS increase in MMCs. PFSE treatment notably decreased the ROS increase in a dose-dependent manner. HG significantly decreases the activity of SOD and GSH are observed (*P* < 0.01 and *P* < 0.001, respectively), as shown in Figure 2B,C. Treatment of PFSE increased the SOD activity and GSH levels in cells. In addition, DPI and Met also significantly inhibited the intracellular ROS increase and stimulated antioxidant enzymes (Figure 2B). We further investigated an effect of PFSE on NOX activity in MMCs, as shown in Figure 2D, the PFSE treatment. The NOX activity was significantly increased under hyperglycemic conditions, and the PFSE treatment abolished the NOX activity from 50 μg/mL. In addition, the NOX inhibitor and AMPK activator also inhibited the activity. These data suggest that PFSE shows significant antioxidative effects against HG-induced oxidative stress in MMCs.

### 3.3. PFSE Regulates HG-Induced NOX and AMPK Activation

**Figure 3:**
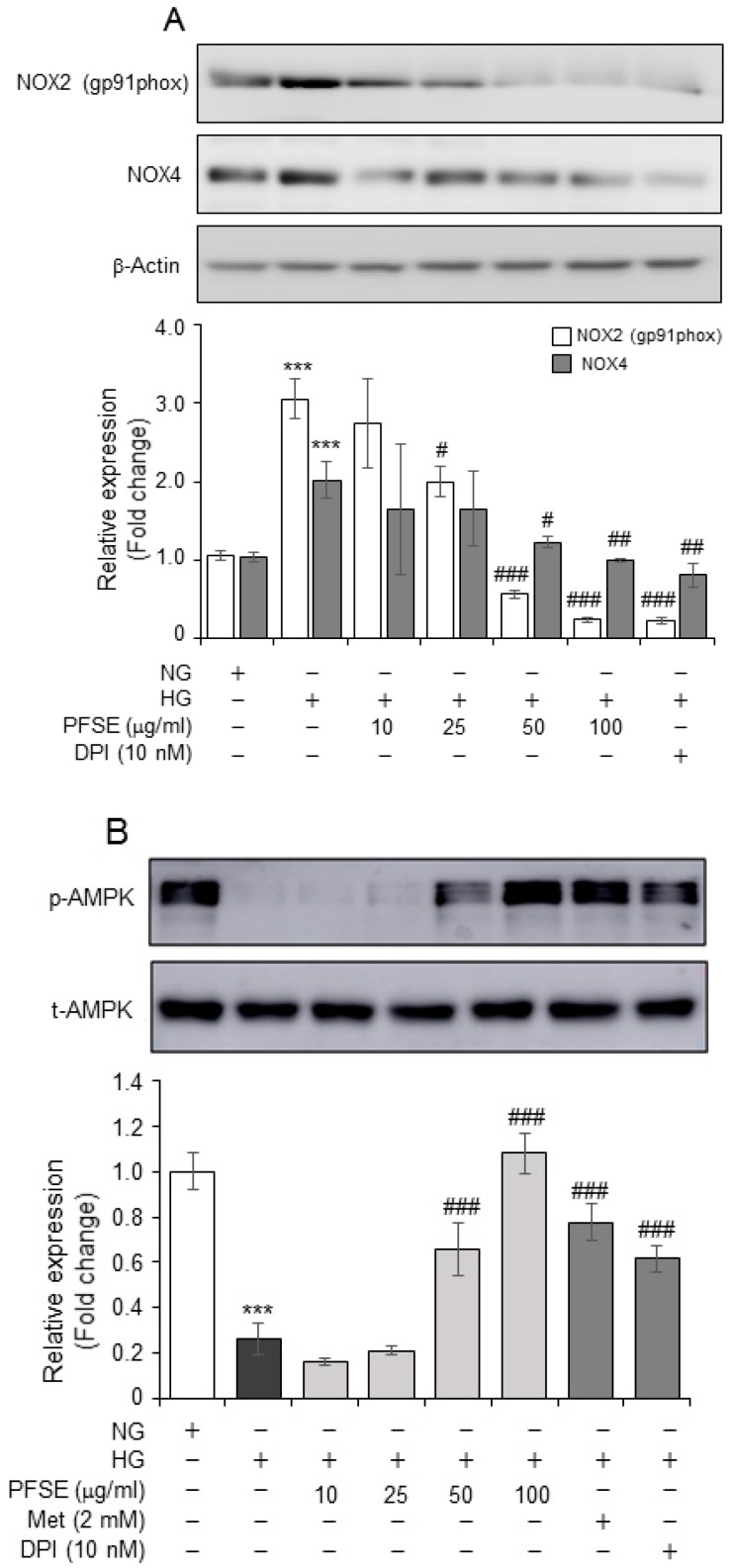
HG-mediated NADPH oxidase (NOX) overexpression and AMP-activated kinase (AMPK) dephosphorylation was inhibited by PFSE. (**A**) The protein levels of NOX2 and NOX4 after 48 h of HG treatment were determined by immunoblotting. PFSE (10, 25, 50, and 100 μM) pretreatment blocked HG-induced NOX protein overexpression. Relative expression of each proteins corresponding to β-actin was presented in the lower panel. (**B**) The effect of varying concentration of PFSE on AMPK phosphorylation was assessed by Western blot. Densitometric data of p-AMPK relative to t-AMPK are presented in lower panel. *** *P* < 0.001 vs. NG; ^#^ *P* < 0.05, ^##^ *P* < 0.01, ^###^ *P* < 0.001 vs. HG. Values are expressed as means ± SEM of three independent experiments.

We next evaluated the molecular mechanisms, which are responsible for the protective effects of PFSE. Since the NOX family have demonstrated that they produce the majority of the intracellular ROS increase in renal injury [5,8], we investigated the effects of PFSE on NOXs activation in MMCs. The expression of NOX2 (gp91phox) and NOX4, as a major source for ROS in the kidneys, was significantly up-regulated in MMCs under the HG conditions. This was ameliorated by PFSE in a dose dependent manner (Figure 3A). As presented in Figure 1, pretreatment with Met (2 mM) dramatically blocked the proliferation and hypertrophy of MMCs in HG conditions. To evaluate the role of PFSE with respect to AMPK activation, PFSE was treated to cells in HG conditions in MMCs. The levels of pAMPK decreased markedly in MMCs under a HG condition, and this was reversed by the pretreatment of MMCs with PFSE from a 50 μg/mL concentration. Met and DPI also reserved HG-induced AMPK phosphorylation (Figure 3B). These results suggest that PFSE blocked HG-induced proliferation and that the protein synthesis of mesangial cells via NOX-induced ROS increases inhibition, as well as AMPK activation.

### 3.4. PFSE Reversed the Effect of HG on Expression of Extracellular Matrix (ECM) Protein

**Figure 4:**
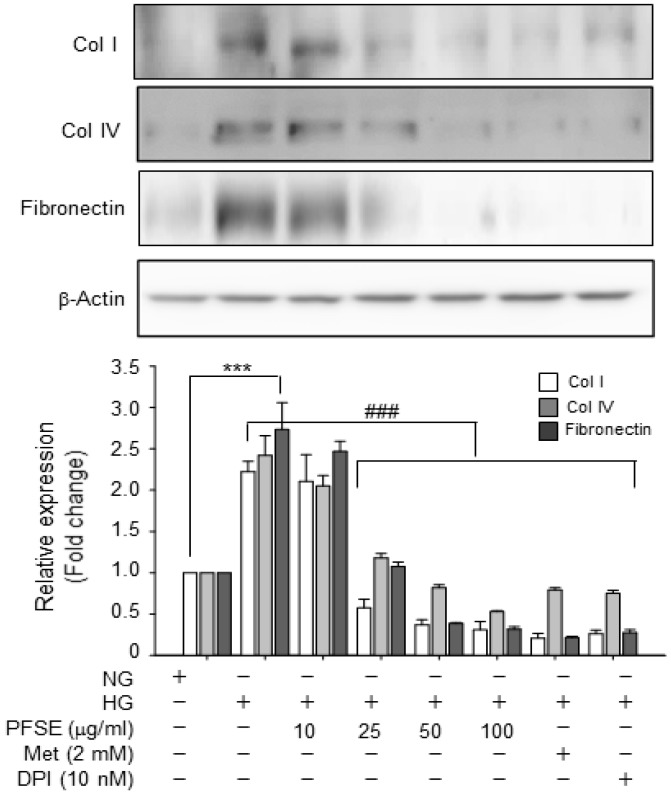
PFSE pretreatment causes a decrease in the extracellular matrix (ECM) protein accumulation in MMCs under HG conditions. Comparison of expression of Col (Collagen) I, Col IV and fibronectin in MMCs. Relative expression of each protein compared with β-actin. *** *P* < 0.001 vs. NG; ^###^ *P* < 0.001 vs. HG. Values are expressed as means ± SEM of three independent experiments.

Next, the efficacy of PFSE on HG-induced fibrosis was evaluated by determining the protein expression of kidney fibrosis-related proteins. Increased synthesis of ECM proteins; such as type I and IV collagen (Col I and Col IV) and fibronectin, are regarded as the makers of proliferative nephritis [3,17]. HG significantly increased the protein expression of Col I and IV and fibronectin in MMCs (*P* < 0.001), which was remarkably abrogated by the PFSE pretreatment with 25~100 μg/mL (*P* < 0.001) (Figure 4). The findings presented here demonstrated that PFSE could also inhibit HG-induced ECM production in MMCs.

## 4. Discussion

**Figure 5:**
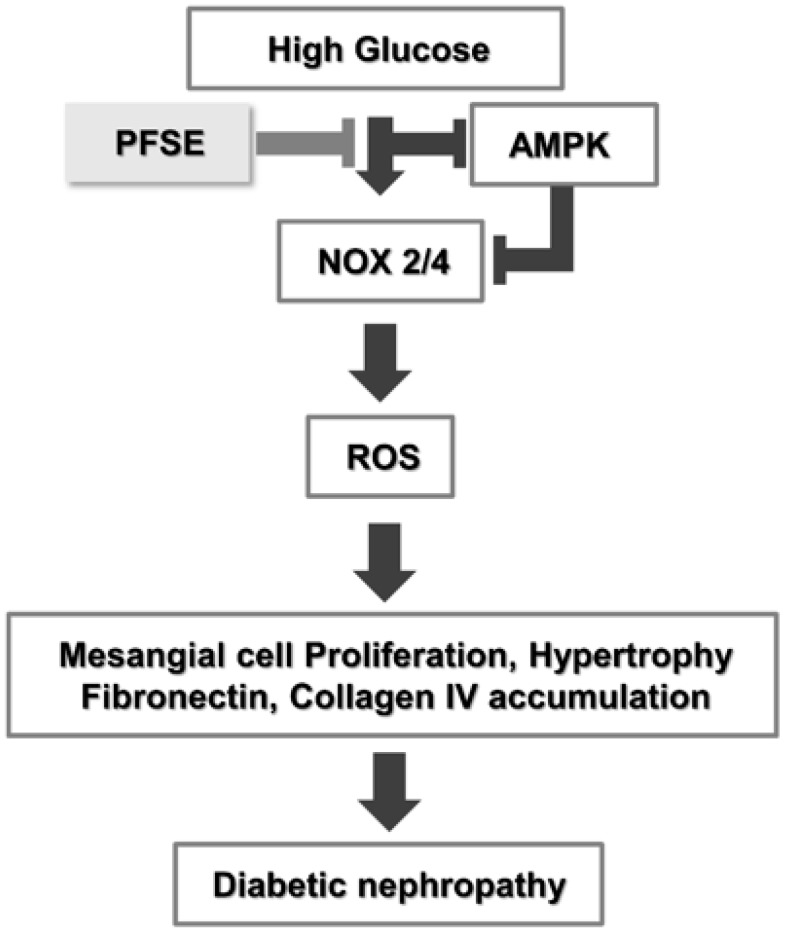
PFSE protects HG-mediated mesangial cell damage via NADPH oxidase and AMPK signaling regulation. High glucose stimulates NADPH oxidases activation and reduces AMPK activity in mesangial cells. Upon activation, NADPH oxidase leads to a ROS increase, endogenous antioxidant’s reduction, and stimulate mesangial cell damage. PFSE may protect against HG-induced proliferation, hypertrophy, and ECM protein accumulation by inhibiting this pathway.

Our previous studies have suggested that PFSE have the potential to protect against the development of diabetes via modulation of the AMPK pathway [13]. The effective concentration was higher than 100 μg/mL in HepG2 cells. DN is the major cause of chronic renal failure in diabetes mellitus (DM) [1,18]. Previously, we also reported that RA was the major compound in PF sprout [13]. Studies reported that RA exerts the renal protective role to DN and has antioxidant and anti-inflammatory activity [19,20,21]. However, whether the PF sprout can attenuate renal damage has not been determined. In this study, we demonstrated that PFSE is potent in reducing high glucose-mediated murine mesangial cells proliferation and fibrosis, by regulating AMPK and NOX-derived ROS generation with an effective concentration of 25~100 μg/mL (Figure 5).

DM is associated with an increase in the generation of ROS, in the kidney, which is involved in glomerulosclerosis. NOX is the major sources of ROS in renal cells. NOX catalyzes the production of O_2_^-^ by the non-electron reduction of O_2_ using NAD(P)H as the electron donor. NOX hyperactivation leads to excessive ROS generation that disrupts the redox network, which is normally regulated by antioxidant systems. This results in oxidative stress, which triggers molecular processes that contribute to tissue injury. Several NOX families have been identified, including NOX1-7, of which NOX1, 2, and 4 activity are altered by diabetes, or diabetic complications. The most extensively studied NOX isoform is the phagocyte NOX2 (gp91phox), which requires regulatory subunits such as the small transmembrane protein p22phox, and the cytosolic regulatory subunits p47phox and p67phox [22]. Unlike all the other NOX proteins, NOX4 is constitutively active and is independent of cytosolic activator proteins or regulatory domains. Among the NOX families, NOX2 and NOX4 have been identified as involved in HG-dependent oxidative stress and fibronectin or Col IV accumulation in MCs [23,24]. SOD is the major antioxidant enzyme, and it converts superoxide into hydrogen peroxide and molecular oxygen [25]. GSH, a well-known defensive antioxidant and a cofactor of glutathione peroxidases, is capable of preventing ROS-induced cellular damage [26]. In our study, PFSE treatment inhibited HG-induced ROS generation as well as prevented SOD inactivation and GSH reduction in MMCs (Figure 2). The NOX2 and NOX4 overexpression was also attenuated by the PFSE treatment under hyperglycemic conditions in MMCs (Figure 3A).

AMPK is a ubiquitously expressed heterotrimeric kinase, which is a regulator of cellular energy homeostasis. AMPK consists of an α, β, and a γ subunit and is activated via phosphorylation of threonine reside 172 [27,28]. AMPK is expressed in the kidney, where it is involved in the diverse physiological and pathologic processes, including ion transport, podocyte function, and diabetic renal hypertrophy [29]. In addition, a recent study that AMPK phosphorylation was also reduced by nearly 70% in the renal cortex of *db*/*db* mice, a model of T2DM [30]. AMPK has also been closely linked to fibrosis promoting pathways. Several groups reported that AMPK activation was able to reduce mesangial matrix expansion and urinary TGF-β1 level as well as inhibit glomerular collagen and fibronectin accumulation in mouse models of diabetic kidney disease [31,32,33].

The classical pathways involved in the generation of advanced glycosylation end products, the activation of protein kinase C, and the aldose reductase pathway, contribute to the development of DN, in which oxidative stress appears to be a common result. In this regard, targeting AMPK in DN could mitigate these adverse effects by modulating glucose-induced oxidative stress metabolism [29].

It was reported that AMPK activation has the potent ability to reduce NOX activation and H_2_O_2_ production [12,32,34]. Whereas, Banskota et al., reported that AMPK phosphorylation was suppressed by NOX2-activaed ROS production in an invasive phenotype response in human colon cancer cells (HT29) [35]. In addition, it has been reported that ROS can directly regulate AMPK activity [36]. Consistent with prior reports, the AMPK activator also inhibited HG-induced oxidative stress in MMCs (Figure 2). Our results also showed that the NOX inhibitor blocked AMPK inactivation in MMCs under hyperglycemic conditions (Figure 3B). Our study revealed that PFSE prevented HG-stimulated AMPK dephosphorylation in MMCs. Finally, PFSE attenuated HG-induced ECM accumulation via downregulation of Col IV and fibronectin protein in mesangial cells (Figure 4).

## 5. Conclusions

In conclusion, the present study provides evidence that PFSE attenuated proliferation and hypertrophy, ROS generation, and inactivation of SOD and reduction of GSH levels in MMCs under hyperglycemic conditions, which may contribute to its renoprotective effects of DN. Interestingly, PFSE prevented HG-induced NOX2 and 4 overexpression and AMPK dephosphorylation in MMCs. PFSE also attenuated ECM, Col IV and fibronectin, protein accumulation in MMCs. These findings suggest that PFSE may serve as one useful new therapeutic agent of DN.

Finally, in pathological conditions related to diabetic nephropathy, there are several associated signaling pathways. Therefore, further studies are required to elucidate the direct and specific mechanisms and in vivo study to provide further evidence to support its therapeutic potential.
